# Increased brain uptake of pterostilbene loaded folate modified micellar delivery system

**DOI:** 10.1080/10717544.2022.2126559

**Published:** 2022-09-21

**Authors:** Yinan Wang, Yanan Su, Yunqiao Yang, Huan Jin, Moli Wu, Qian Wang, Pengyuan Sun, Jianbin Zhang, Xiaobo Yang, Xiaohong Shu

**Affiliations:** a Institute of Integrative Medicine, Dalian Medical University, Dalian, China; b College of Pharmacy, Dalian Medical University, Dalian, China; c The First Affiliated Hospital of Dalian Medical University, Dalian, China; d College of Basic Medical Sciences, Dalian Medical University, Dalian, China

**Keywords:** blood-brain barrier, pterostilbene, micelles, folate, receptor-mediated endocytosis, drug targeting index

## Abstract

Effective chemotherapy for clinical treatment of brain diseases is still lacking due to the poor penetration of the blood-brain barrier (BBB). The aim of this study was to construct a folate modified pterostilbene (Pt) loaded polymeric micellar delivery system (F-Pt/M) with mPEG-PCL as carrier material to aim at penetrating the BBB for brain tissue targeting via receptor-mediated endocytosis. In this study, F-Pt/M was prepared using thin-film hydration method and then optimized by response surface methodology (RSM) with the entrapment efficiency (EE), drug loading (DL) and hydrodynamic diameter (HD) as indexes. The average hydrodynamic diameter and zeta potential of optimal F-Pt/M were 133.2 nm and 24.6 mV, respectively. DL (18.3%) and EE (98.6%) made the solubility of Pt in water about 25 times higher than that of crude Pt. Results of DSC evaluation revealed that drugs were successfully encapsulated inside the polymeric micelles. TEM images showed that homogeneous spherical micellar structures with a narrow size distribution were developed. The release result in vitro showed that F-Pt/M presented sustained release behavior compared to control free Pt solution. Compared to non-targeted Pt/M, F-Pt/M had a significantly higher cytotoxicity against FR-overexpressing A172 cells. In vitro cellular uptake tests illustrated that the micellar delivery system could significantly improve the accumulation of drugs in target cells via receptor-mediated endocytosis. BBB penetration value (P) of F-Pt/M was about 4 folds higher than that of free Pt group. In addition, drug targeting index (DTI) was calculated to determine targeting of F-Pt/M to the brain which was found to be 4.89, implying improved brain targeting was achieved. Hence, the developed F-Pt/M exhibited great potential for delivering more drug molecules across the BBB for the treatment of brain diseases.

## Graphical Abstract

**Figure:**
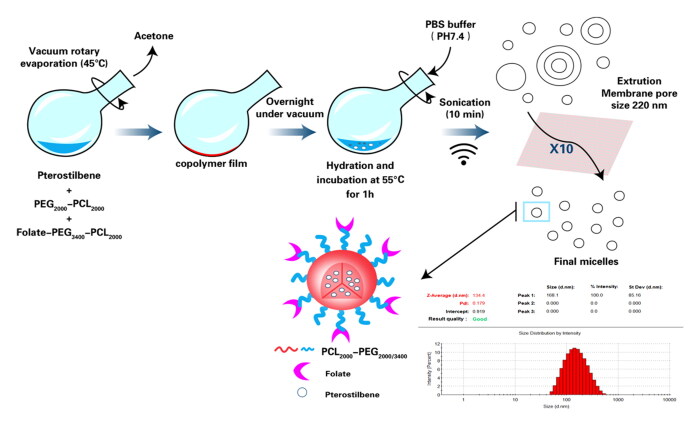

A novel folate modified pterostilbene (Pt)-loaded polymeric micelle (F-Pt/M) drug delivery system, composing of Pt (as model drug), folate (as targeting ligand) and PEG2000/PEG3400-PCL2000 (as self-assembled material), was prepared by thin-film hydration method. The developed F-Pt/M enhanced the penetration of Pt across the blood-brain barrier via receptor-mediated endocytosis. It might have the potential to be developed as an attractive micellar system for effective chemotherapy against some brain diseases.

## 1. Introduction

Brain diseases, such as brain cancers, Huntington’s disease, Alzheimer disease and Parkinson’s disease, are still poorly treated diseases. Despite significant recent improvements, drug development for brain diseases still has the poorest success rates in comparison with other therapeutic areas due to the complexity of the brain, multi-drug resistance, side effects and especially the impermeable blood brain barrier (BBB) (Lingineni et al., 2017). The BBB, a functional and anatomic barrier, impedes the entrance of toxic substances from the bloodstream and regulates homeostasis of the CNS microenvironment. The transport of therapeutic agents across the BBB is exactly constrained by both physical adherent junctions, tight junctions and a variety of transport systems, such as ATP-binding cassette transporters. As known, less than 2% of small molecules (only lipid soluble molecules with a molecular weight <400 Da) and no large molecules are able to bypass the BBB (Pardridge, 2003; Dong, 2018). Nowadays, intra-cerebral injection, intra-cerebroventricular injection and convection-enhanced diffusion are usually employed to treat CNS disorders in clinical therapy. Inevitably, these approaches have brought great inconvenience and pain to patients (Pardridge, 2007). Therefore, development of effective strategies to deliver more drug molecules to the CNS is urgently needed for the treatment of brain diseases.

To achieve sufficient drug delivery across the BBB, numerous strategies have been developed. Nanoscale drug delivery systems, for example, various nanoparticles, micelles, hydrogels and liposomes, are an impressive noninvasive method to deliver drugs into brain (Khan et al., 2016; 2018; Ruan et al., 2021). In recent years, great efforts have been made to develop polymeric micelles, a kind of nano-sized carriers composed of amphiphilic block copolymers that can self-assemble into hydrophobic core-hydrophilic shell structures, which have been evaluated under several clinical trials as vehicles for anticancer drugs (Nakanishi et al., 2001; Gothwal et al., 2016). Polymeric micelles are considered to be an excellent drug delivery system for hydrophobic therapeutic agents. Due to ease of functionalization, they can provide targeting ability, improve solubility, increase tissue penetration, reduce toxicity, prolong the circulation time, and so on (Kalhapure et al., 2015; Matsumoto et al., 2016; Raval et al., 2021). Methoxy poly(ethylene glycol)(MPEG) block PCL (MPEG-PCL) is an ideally suitable copolymer because of it being nontoxic, biocompatible and biodegradable (Kheiri Manjili et al., 2017a). Several studies have demonstrated that MPEG-PCL micelles can significantly improve the drug’s water solubility, control drug release, prolong the mean residence time of drugs, avoid them being captured by macrophages and show great potential for clinical treatment (Wei et al., 2009; Guo et al., 2021; Shahid et al., 2021; Luo et al., 2022).

Specific ligands or substrates have been extensively employed to enhance the efficiency of BBB penetrating and brain-targeting delivery by binding with specific carrier-mediated transportation or receptor-mediated transcytosis on the BBB (Gao, 2017; Han & Jiang, 2021). Folate or folic acid (FA), a B vitamin that plays a fundamental role in DNA synthesis, is considered an essential nutrient for both healthy and tumoral cells. The cell uptake of FA could be mediated by folate receptors (FR) (Jain & Jain, 2015). Since tumoral cells have an aggressive duplication rate, the FRs are commonly overexpressed on the membranes to ensure the supply of FA for the cell proliferation process. It is said that FR is overexpressed around 500-fold higher in tumoral cells than in normal cells, which facilitates its application as a promising strategy to target nanoscale drug delivery systems to tumoral cells, such as glioma cells (Fernandez et al., 2018; Kucheryavykh et al., 2019; McCord et al., 2021). Additionally, as FA is essential to the CNS, there are FRs in the BBB, which makes it possible to deliver FA conjugated nano-carriers through the BBB (Luiz et al., 2021). Thus, as an active targeting ligand, FA could be potentially employed for transporting drugs through the BBB and targeting brain tumor cells (Kuo et al., 2019; Lu et al., 2019).

Pterostilbene (3,5-dimethoxy-4′-hydroxy-trans-stilbene, Pt), a dimethylether analog of resveratrol, has similar chemical, physiological and pharmacological properties to resveratrol. Although the poor solubility of Pt limits its application (Zhang et al., 2014), it has been extensively studied in recent years. It has been reported that Pt has a wide range of pharmacologic effects such as anti-oxidant (Amarnath Satheesh & Pari, 2006), neuroprotective (Chang et al., 2012), anti-tumor (Ma et al., 2019), anti-inflammatory (Liu et al., 2016) and hypoglycemic (Bhakkiyalakshmi et al., 2014) activities with low or no significant toxicity according to data from preclinical and clinical trials (Ruiz et al., 2009; Riche et al., 2013). Therefore, as a promising and potential anticancer and neuroprotective compound, it is expected to become a candidate drug in clinical treatment of brain diseases due to its efficacy and safety.

Although various delivery systems have been developed for Pt delivery, such as nanoparticles (Romio et al., 2018; Zhao et al., 2021; Zou et al., 2021; Wei et al., 2022), nanoemulsions (Zhang et al., 2014; Liu et al., 2019), micelles (Silva et al., 2014; Song et al., 2020) and inclusion complexes (Silva et al., 2014). To our knowledge, there have been no studies developing brain targeting Pt delivery systems. Considering the advantages of polymeric micelles as drug carriers and the potential of Pt as a chemotherapeutic agent for the treatment of brain diseases, we developed folate modified Pt loaded micelles (F-Pt/M) for the purpose of facilitating the transmigration of Pt across the BBB as well as its therapeutic efficacy in the treatment of related diseases. This study’s aim was to aid in the determination of the potential of F-Pt/M as a nanoscale drug delivery system with the ability to penetrate the BBB and target brain tissues.

## 2. Materials and methods

### 2.1. Materials

Pterostilbene (>99%) was purchased from Baoji Herbest (Shanxi, China). PEG_2000_-PCL_2000_ and Folate-PEG_3400_-PCL_2000_ were obtained from Xi’an Ruixi (Shanxi, China). Dimethyl sulfoxide (DMSO, ≥99.9%) and coumarin-6 were purchased from Sigma Aldrich (USA). Cell Counting Kit-8 (CCK-8) was purchased from Nanjing KeyGen (Jiangsu, China). Phosphate buffered saline (PBS) (Beijing Solarbio Science & Technology, China) was employed in the study. All other reagents used were of analytical grade.

### 2.2. Animals

Male SD rats weighing 200–250 g (Experimental Animal Center of Dalian Medical University, China) were maintained in standard conditions on a 14 h light/10 h dark cycle provided with food and water ad libitum prior to experimental procedures. All animal experimental protocols were conducted with an approval by the Institutional Animal Care and Use Committee at Dalian Medical University, Dalian, China (Ethics Committee approval number: SCXK 2018-0003) as well as in accordance with the recommendations and policies of the National Institutes of Health (NIH) guide for the care and use of laboratory animals (NIH Publications No. 8023, revised 1978).

### 2.3. Cell line and cell culture

Human glioblastoma A172 cells (Nanjing KeyGen Biotech Co., Ltd., Nanjing, China) which overexpress folate receptor (Weitman et al., 1992) were used. Cells were routinely cultured in DMEM medium (Invitrogen, Grand Island, NY, USA) supplemented with 10% fetal bovine serum (Gibco Life Science, Grand Island, NY, USA) and 1% streptomycin/penicillin in a humidified atmosphere of 5% CO_2_ at 37 °C. Cell passages were carried out according to the manufacturer’s instructions.

### 2.4. Preparation of folate modified pterostilbene loaded PEG_2000_/PEG_3400_-PCL_2000_ micelles

**Figure 1.**
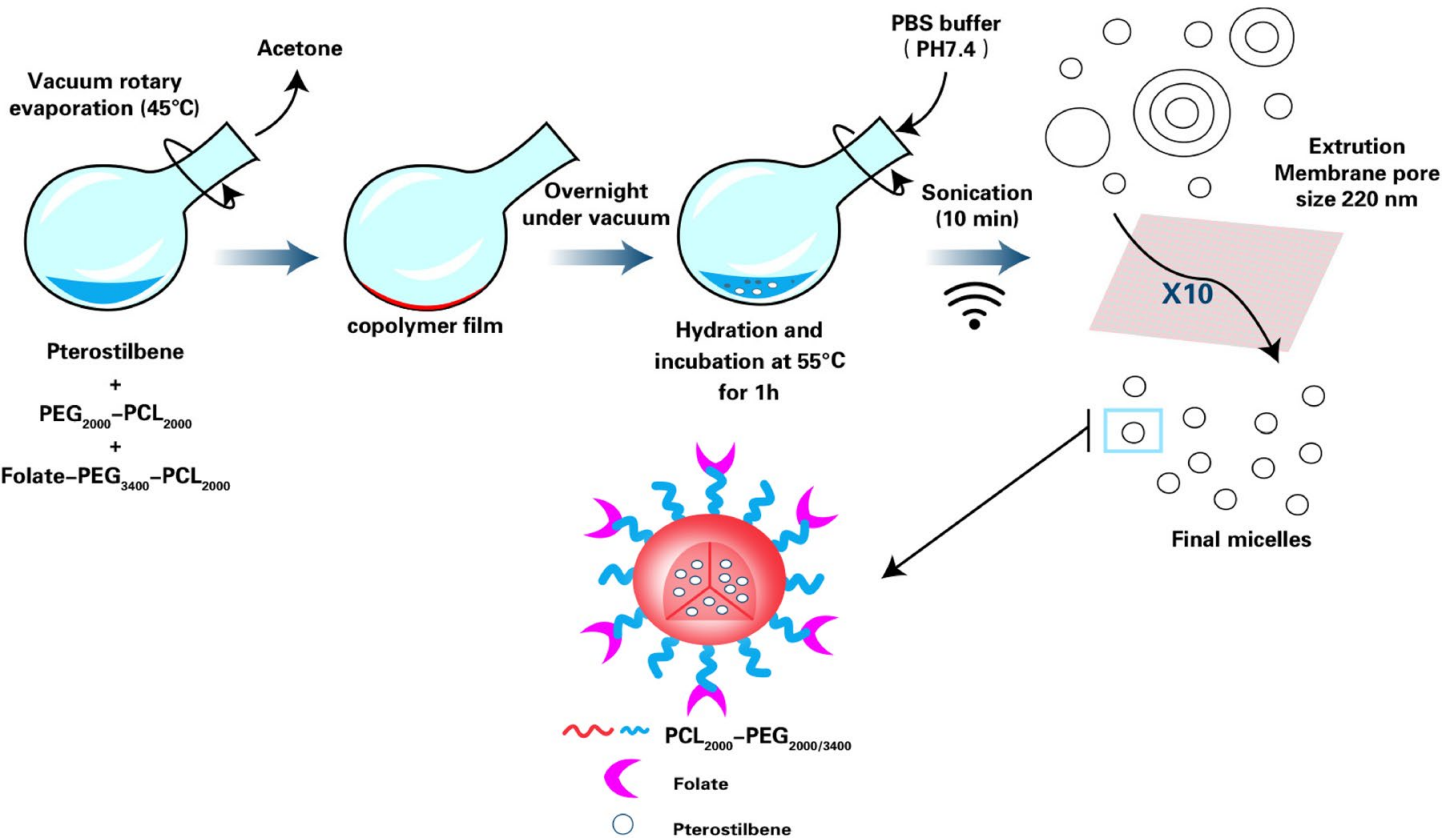
Schematic representation for the preparation of folate modified pterostilbene loaded micelles by thin-film hydration method.

Folate modified pterostilbene loaded PEG_2000_/PEG_3400_-PCL_2000_ micelles (F-Pt/M) were formulated using a thin-film hydration method (Zheng et al., 2015; Mei et al., 2019). In brief (see Figure 1), Pt, PEG_2000_-PCL_2000_ and Folate-PEG_3400_-PCL_2000_ of various concentrations and proportions were dissolved in acetone in a round-bottom flask. The organic solvent was removed at 45 °C by vacuum rotary evaporation to form a dry film at the bottom of the flask. The obtained film was further dried overnight under vacuum to remove any traces of remaining acetone. After adding the preheated PBS (55 °C) to hydrate the film, the system was vortexed and sonicated for 10 min, respectively. Free unentrapped Pt was removed via using centrifugation method. Separation was carried out by centrifuging at 10,000 rpm for 10 min (Centrifuge H1650-W, Xiangyi, Hunan). Finally, the supernatant was extruded through a polycarbonate membrane of 220 nm pore size for 10 times using a laboratory extrusion device (LiposoFast Basic LF-1, Avestin Inc., Ottawa, Canada). In addition, blank micelles (BM) and pterostilbene loaded micelles (Pt/M) were prepared via the same way. To study the uptake of folate modified micelles by cancer cells, the coumarin-6 (C6), a fluorescent material, loaded micelles (C6/M and F-C6/M) were prepared as well.

### 2.5. Experimental design

After preliminary screening studies, two independent variables, namely amount of pterostilbene (X_1_) and volume of hydration PBS solution (X_2_) in the preparation of polymeric micelles, were chosen to optimize formulation using three dependent responses including entrapment efficiency (EE), drug loading (DL) and hydrodynamic diameter (HD) as criteria. A two-factor five-level CCRD-response surface method (Design Expert, Version 8.0.6, Stat-Ease Inc., Minneapolis, MN) which dictated thirteen experimental runs was employed to derive the optimum levels of the independent variables. The levels of each independent variable were designated as +1.414, +1, 0, −1 and −1.414, respectively, and the corresponding actual values for each variable are listed in Table 1. The following polynomial equation was fitted to the data:

(1) $$Y\;={\;b}_{0}+{\;b}_{1}X_{1}+{\;b}_{2}X_{2}+{\;b}_{12}X_{1}X_{2}+{\;b}_{11}X_{1}{}^{2}+{\;b}_{22}X_{2}{}^{2}$$

**Table 1.** Central composite design with code values and observed response values (*n* = 3, mean ± SD).

No.	Code values of variables		Actual values of variables		Responses		
	X_1_	X_2_	Amount of pterostilbene (mg)	Volume of hydration solution (mL)	Y_EE (%)_	Y_DL (%)_	Y_HD (nm)_
1	0	0	11	9	68.7 ± 1.0	17.2 ± 0.3	138.2 ± 3.6
2	−1	1	4.64	13.24	95.1 ± 4.3	11.7 ± 3.0	81.0 ± 2.9
3	1	1	17.36	13.24	68.5 ± 1.5	22.1 ± 1.5	169.2 ± 5.1
4	0	−1.414	11	3	25.9 ± 0.4	6.5 ± 0.1	132.3 ± 5.6
5	1.414	0	20	9	28.0 ± 0.3	10.6 ± 0.1	132.7 ± 5.4
6	−1.414	0	2	9	90.8 ± 1.4	5.2 ± 0.1	74.5 ± 2.3
7	−1	−1	4.64	4.76	72.9 ± 1.0	9.0 ± 0.1	85.4 ± 2.0
8	0	0	11	9	68.8 ± 0.4	17.2 ± 0.1	138.3 ± 2.3
9	0	0	11	9	67.7 ± 0.2	16.9 ± 0.1	134.2 ± 2.0
10	1	−1	17.36	4.76	25.8 ± 0.3	8.9 ± 0.1	117.0 ± 2.7
11	0	0	11	9	67.8 ± 0.7	17.0 ± 0.2	134.2 ± 1.2
12	0	1.414	11	15	90.3 ± 1.1	22.6 ± 0.3	150.1 ± 2.0
13	0	0	11	9	59.0 ± 1.3	14.8 ± 0.2	147.9 ± 1.5

Where Y, the response associated with each factor level combination, b_0_, the intercept and b_1_–b_22_, the regression coefficients of the factors.

### 2.6. Characterization of folate modified pterostilbene loaded PEG_2000_/PEG_3400_-PCL_2000_ micelles

#### 2.6.1. Hydrodynamic diameter and zeta potential

The intensity-weighted hydrodynamic diameter and size distribution of polymeric micelles were determined by laser light scattering technique (LLS) (Nano-2S 90 Zetasizer, Malvern, UK) at room temperature. The size distribution was given by polydispersity index (PDI, a value between 0 and 1). The surface charge of micelles was measured by electrophoretic light scattering technique on a zeta potential analyzer (Nano-2S 90 Zetasizer, Malvern, UK). All samples were measured in triplicate (*n* = 3).

#### 2.6.2. Transmission electron microscopy (TEM)

The morphologies of polymeric micelles were observed using transmission electron microscopy (Jeol JEM-2000EX, Tokyo, Japan). The samples were negatively stained by 4% phosphotungstic acid and dried in air on carbon-coated grids before observation.

#### 2.6.3. Differential scanning calorimetry (DSC) studies

Differential scanning calorimeter (DSC60, Shimadzu Co., Ltd., Tokyo, Japan) was used to record DSC thermograms of samples (Pt, PEG_2000_-PCL_2000_, Folate-PEG_3400_-PCL_2000_, lyophilized Pt/M and lyophilized F-Pt/M). Samples were heated in an aluminum pan at a rate of 10 °C/min in an atmosphere of nitrogen to 300 °C and thermograms were recorded (Yang et al., 2011).

#### 2.6.4. Entrapment efficiency (EE) and drug loading (DL)

In order to measure the EE and DL of F-Pt/M, the drug loaded micelles were dissolved in ethanol, vortexed for 1 min and then sonicated for 10 min to destroy the structure of micelles. The absorbance of resultant solutions was determined by spectrophotometer (Thermo Scientific Inc., Finland) with excitation wavelength at 330 nm and emission wavelength at 374 nm. The EE and DL of F-Pt/M were calculated according to the following equations,

(2) EE (%) = (weight of Pt in micelles)/(weight of feeding Pt) × 100

(3) $$\text{DL}\;\left(\%\right)\;=\;\left(\text{weight}\;\text{of}\;\text{Pt}\;\text{in}\;\text{micelles}\right)/\left(\text{weight}\;\text{of}\;\text{Pt}\;\text{in}\;\text{micelles}\;+{\;\text{weight}\;\text{of}\;\text{feeding}\;\text{PEG}}_{2000}-{\text{PCL}}_{2000}\text{and}\;\text{Folate}-{\text{PEG}}_{3400}-{\text{PCL}}_{2000}\right)\;\times \;100$$

### 2.7. In vitro drug release

With free Pt acetone solution as control, in vitro release of Pt from F-Pt/M formulation was studied by the dialysis method (Khan et al., 2020; Rubab et al., 2021; Yang et al., 2021). Briefly, 1 mL of samples, equivalent to 0.8 mg drug, were filled into dialysis bags (molecular weight cutoff: 3500 Da) and dialyzed against 1,000 mL of phosphate buffer solution of pH values 7.4 (containing 0.3% Tween 80) to mimic drug release in normal tissues/blood. At the desired time intervals, 1 mL of release medium was withdrawn and replaced with 1 mL of fresh medium. The amount of released Pt was measured by spectrophotometer (Thermo Scientific Inc., Finland) with excitation wavelength at 330 nm and emission wavelength at 374 nm.

In order to understand the kinetic model of Pt release from F-Pt/M, zero-order, first-order and Higuchi models were fitted, and the highest adjusted R^2^ value was used as the criterion for model selection.

### 2.8. In vitro cytotoxicity and cellular uptake

#### 2.8.1. In vitro cytotoxicity assay

To evaluate the inhibitory effects of Pt in various formulations on A172 cells, cytotoxicity assay was carried out with CCK-8 kits. Cells were seeded into 96-well plates at a density of 5,000 cells per well, followed by incubation for 24 h. Cells were treated with free Pt solution (free Pt), Pt loaded micelles (Pt/M), folate modified Pt loaded micelles (F-Pt/M) and blank micelles (BM) at equivalent drug concentrations ranging from 5 μM to 100 μM and further incubated for 48 h. Then, CCK-8 reagent (10 μL) was added to each well. After 2 h of incubation at 37 °C, the absorbance intensity was measured by a microplate reader (Varioskan LUX; Thermo Scientific Inc., Vantaa, Finland) at 450 nm. Cell viability was calculated by the ratio between the fluorescence absorbance of the cells treated with various Pt formulations (or blank micelles) and that of the cells incubated with the culture medium only. The IC_50_ values were calculated by using SPSS 22.0 (IBM Corp., Armonk, NY).

#### 2.8.2. In vitro cellular uptake assay

In view of the hydrophobicity of Pt, the fluorescent probe coumarin-6 (C6) was utilized as a similar substitution of Pt to observe cellular uptake (Dong & Feng, 2007) and incorporated into C6 loaded polymeric micelles (C6/M) and folate modified F-C6/M with similar preparation procedures as Pt/M or F-Pt/M formulations. The glioblastoma A172 cells were selected for cell uptake experiment.

For the fluorescence microscopy study, A172 cells were seeded on cover slips placed in 24-well plates (1 × 10^4^ cells/well) and grown for 24 h at 37 °C to allow cell adhesion and spread. Then, the cells were treated with free C6 solution, C6/M or F-C6/M (100 ng/mL of C6) for 4 h. Subsequently, the medium was removed and cells were rinsed thrice with cold PBS followed by fixing with 4% paraformaldehyde for 30 minutes. The cells were further washed three times with cold PBS and then stained with DAPI at 37 °C for 5 min. The cellular morphology was observed via an inverted fluorescence microscope (Olympus ix83, Olympus Optical Co., Ltd., Tokyo, Japan).

For quantitative cellular uptake analysis, A172 cells were seeded (1 × 10^6^ cells/well) in 6-well plates and cultured for 24 hours to encourage cell attachment. The medium was changed to the suspension of free C6, C6/M or F-C6/M (100 ng/mL of C6). After 1 h and 4 h incubation, the wells were washed three times with cold PBS to remove the C6 outside the cells. Then, cells were trypsinized, pelleted by centrifugation, washed thrice with PBS and finally lysed by RIPA Lysis Buffer. Spectrophotometer (Varioskan LUX; Thermo Scientific Inc., Vantaa, Finland) was used to measure the fluorescence intensity of C6 with excitation wavelength at 456 nm and emission wavelength at 500 nm.

Additionally, a competitive group was designed to study the competition effect of folate on the cellular uptake. An excess of free folate (10 μg/mL) was added to the culture medium 0.5 h prior to the addition of F-C6/M. All other steps were in the same way as above described.

### 2.9. In vitro hemolysis assay

The hemocompatibility of F-Pt/M was investigated by hemolysis assay (Valenzuela-Oses et al., 2017). Fresh SD rats blood was diluted by physiological saline, and red blood cells (RBCs) were collected by centrifugation at 1,200 rpm for 5 min. Following carefully washing and diluting, RBC suspension in normal saline (v/v, 2%) was prepared for hemolysis study. Pt loaded F-Pt/M and free Pt at different drug concentrations were added and mixed by vortex. Then, the samples were incubated at 37 °C for 1 h and after that were centrifuged at 3,000 rpm for 10 min. The supernatant was collected and spectrophotometrically measured on a microplate plate reader (Varioskan LUX, Thermo Scientific Inc., Vantaa, Finland) at 540 nm. Double distilled water and normal saline were employed as positive and negative control, respectively. The hemolysis ratio (HR) of RBCs was calculated using the following equation:

(4) $$\text{HR}\;\left(\%\right)\;=\;\left(A_{s}–{\;A}_{0}\right)/\left(A_{100}–{\;A}_{0}\right)\;\times \;100$$

Where, A_s_ is the absorbance of sample, A_0_ is the absorbance of negative control and A_100_ is the absorbance of positive control.

### 2.10. Brain targeting and brain distribution study in rats

#### 2.10.1. Sample collection and extraction procedure

Rats were randomly divided into two groups of 24 animals each. Each group received either freshly prepared control free Pt solution in DMSO (10%) in saline solution or formulation of F-Pt/M at a dose equivalent to 3 mg/kg of drug intravenously through lateral tail vein. From each group, three rats were sacrificed at intervals of 5, 15, 30, 45, 60, 90, 120 and180 min post dose. At each time point, blood sample was withdrawn and centrifuged at 3,500 rpm at 4 °C for 10 min. At the same time, the brain sample was harvested, weighed, and homogenized in PBS after cardiac perfusion with 0.9% normal saline solution. Samples were stored at −80 °C until analysis.

Pt from both brain and plasma samples was extracted by liquid-liquid extraction method. Briefly, 20 µL of internal standard working solution (resveratrol, 250 ng/ml) was added to 200 µL aliquot of plasma or brain sample. After 30 seconds of vortexing, 1 mL of extraction solvent, methyl tertiary butyl ether (MTBE) was added. Afterwards, the sample was mixed for 1 min plus centrifugation at 1,000 rpm for 5 min, the supernatant organic layer was carefully transferred and dried using nitrogen gas with gentle heat at 40 °C. The residue was reconstituted with 400 µL of mobile phase and centrifuged at 12,000 rpm for 10 min. The supernatant was transferred to an auto-sampler vial and 10 µL was then injected into the LC-MS/MS system for analysis. An LC-MS/MS analysis method reported by Ferrer (Ferrer et al., 2005) was employed for the determination of Pt concentration.

#### 2.10.2. Brain distribution and brain targeting index

Pharmacokinetic parameters, such as C_max_ (maximum drug concentration), T_max_ (the time to C_max_) and AUC_0-t_ (area under plasma concentration-time curve), were calculated by DAS 2.0 (Drug and Statistics ver 2.0, China). To examine the blood-brain barrier penetration (P) of Pt, the following formula was employed (Yang et al., 2021):

(5) $$P={\text{AUC}}_{\text{brain}}/{\text{AUC}}_{\text{plasma}}$$

For brain targeting evaluation, DTI (drug targeting index) was determined using the following equation (Katekar et al., 2020):

(6) $$\text{DTI}\;=\;{\left({\text{AUC}}_{\text{brain}}/{\text{AUC}}_{\text{plasma}}\right)}_{\text{test}/}{\left({\text{AUC}}_{\text{brain}}/{\text{AUC}}_{\text{plasma}}\right)}_{\text{control}}$$

When DTI value >1, it implies that brain targeting is obvious.

### 2.11. Statistical analysis

Significance between groups was obtained by one-way analysis of variance (ANOVA) followed by a Dunnett post hoc test and the tests were performed using SPSS 11.0 software. A value of *P* < 0.05 was considered statistically significant.

## 3. Results and discussion

### 3.1. Model fitting

**Figure 2.**
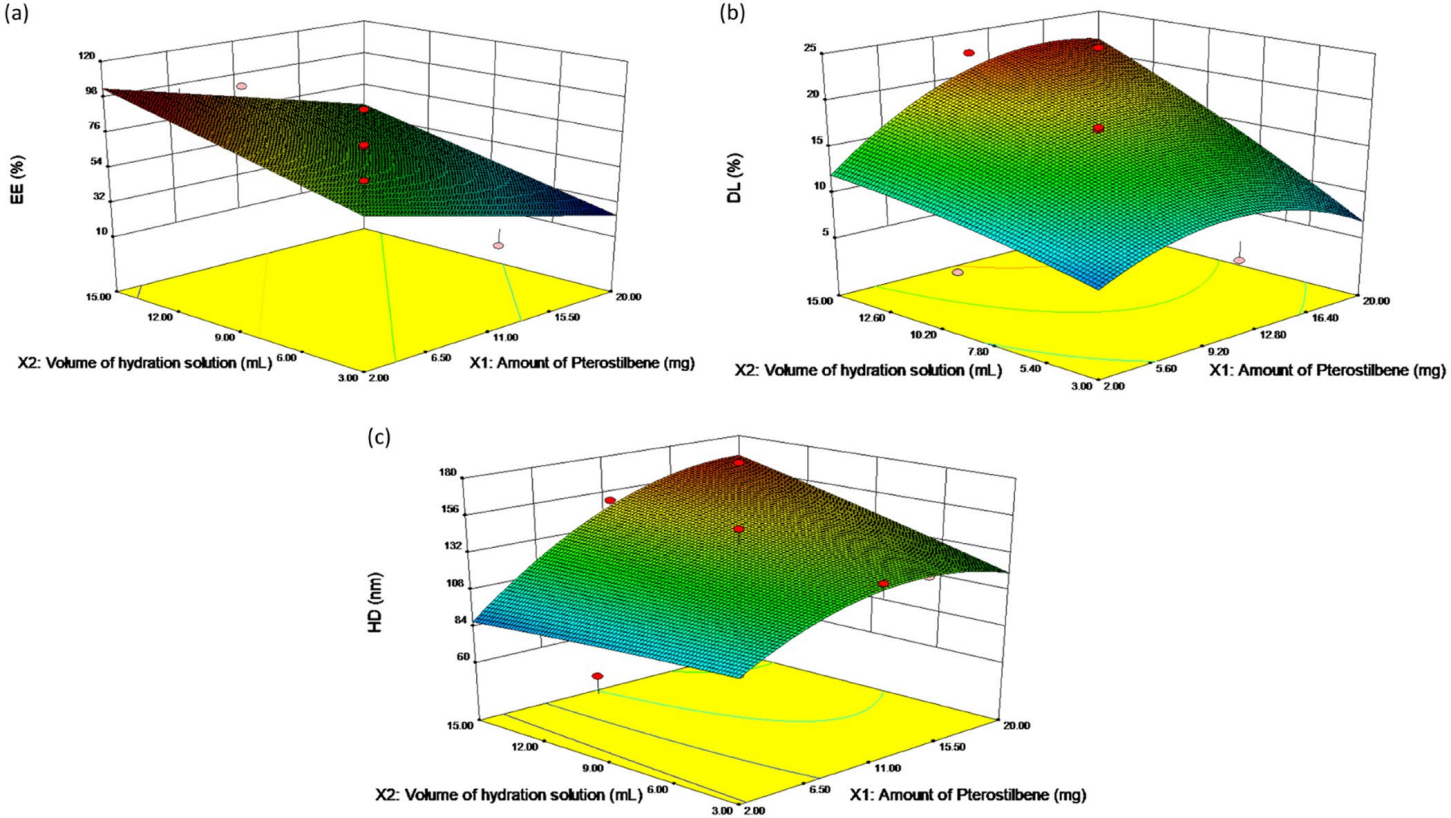
Response surface plots. The effects of amount of pterostilbene and volume of hydration solution on (a) EE, (b) DL and (c) HD. EE, entrapment efficiency; DL, drug loading and HD, hydrodynamic diameter.

In the present study, thirteen experiments were performed in one block. Two independent variables were considered: pterostilbene (X_1_) ranging from 2.0 to 20.0 mg and volume of hydration solution (X_2_) from 3.0 to 15.0 mL. All the formulations were prepared with fixed amounts of PEG_2000_-PCL_2000_ (30.0 mg) and Folate-PEG_3400_-PCL_2000_ (3.0 mg) following the same preparation process. The experimental data of Pt encapsulated polymeric micelles in terms of entrapment efficiency (Y_EE (%)_), drug loading (Y_DL (%)_) and hydrodynamic diameter (Y_HD (nm)_) were presented in Table 1. These considered dependent response variables were analyzed as a raw data in CCRD to deduce its statistical analysis and the best predicted models. The three-dimensional (3 D) response surface plots were also presented according to regression equations (Figure 2).

As shown in Table 1, the EE of Pt micelles ranged from 25.8% to 95.1%. The analysis of variance (ANOVA) (Table 2) revealed that the two independent variables (X_1_ and X_2_) can significantly (*p* < 0.0001) affect the ability of the drug to be encapsulated in the polymeric micelles formed. The adjusted R^2^ value (>90%) indicated a high level of significance for the selected linear model. The regression equation of the fitted model constructed for EE was as follows:

(7) $$\text{EE}\;\left(\%\right)\;=\;57.6\;–\;3.2*X_{1}+\;4.6*X_{2}$$

**Table 2.** Analysis of variance for the fitting models.

Source	Y_EE(%)_				Y_DL(%)_				Y_HD(nm)_			
	*df*	Sum of squares	*F* value	*p* value	*df*	Sum of squares	*F* value	*p* value	*df*	Sum of squares	*F* value	*p* value
Model	2	6343.1	72.1	<0.0001	5	362.9	30.1	0.0001	5	9318.2	24.4	0.0003
X_1_	1	3301.7	75.0	<0.0001	1	40.1	16.7	0.0047	1	5112.0	66.8	<0.0001
X_2_	1	3041.4	69.1	<0.0001	1	187.5	77.9	<0.0001	1	665.1	8.7	0.0215
X_1_X_2_	–	–	–	–	1	27.6	11.5	0.0117	1	800.0	10.5	0.0144
X_1_^2^	–	–	–	–	1	107.6	44.7	0.0003	1	2723.5	35.6	0.0006
X_2_^2^	–	–	–	–	1	2.6	1.1	0.3317	1	7.0	0.1	0.7710
Residual	10	440.0	–	–	7	16.9	–	–	7	535.6	–	
Lack of fit	6	370.7	3.6	0.1195	3	12.7	4.1	0.1047	3	409.9	4.35	0.0948
Pure error	4	69.3	–	–	4	4.2	–	–	4	125.7	–	
Cor total	12	6783.2	–	–	12	379.7	–	–	12	9853.8	–	
R^2^		0.94				0.96				0.95		
Adj-R^2^		0.92				0.92				0.91		
C.V.%		10.4				11.2				7.0		
PRESS		864.2				96.7				3111.4		
Adeq. precision		25.0				15.8				17.3		

For X_1_ (Pt amount) it was found that increasing the amount of Pt within our experimental limits would lead to a decrease in the entrapment ability of the drug (Figure 2(a)), which might be related to the fact that self-assembled PEG-PCL micelles could provide relatively settled spaces to accommodate lipophilic drug molecules; therefore, the EE was chiefly dependent on the number of micelles formed in the system. A similar phenomenon was observed in the development of curcumin loaded phospholipid-sodium deoxycholate-mixed micelles by Yuwei Duan (Duan et al., 2015). Regarding X_2_ (hydration volume), increasing PBS volume was found to increase EE (Figure 2(a)). Sufficient water molecules must exist in the PEG-PCL–water mixture to bind all PEG segments. Increasing the water amount higher than the content of water needed to bind PEG segments will swell only the PEG chains (the PCL chains remain anhydrous). Swelling of the PEG chains gives an increased interface area per PEG chain which would alter the interface curvature (Nour et al., 2016). As a result, the PEG-PCL micelles generally appear as spheres encapsulating more drug molecules.

According to Table 1, the DL of Pt micelles ranged from 5.2% to 22.6%. Polynomial statistical analysis using quadratic model indicated that independent X_1_, X_2_ and polynomial term X_1_X_2_, X_1_^2^ were significant model variables with p-values of <0.05 (Table 2). The final equation in terms of actual variables with a high corresponding adjusted R^2^ value (>90%) was presented as follows:

(8) $$\text{DL}\;\left(\%\right)\;=\;–2.4\;+\;1.6*X_{1}+\;0.7*X_{2}+\;0.1*X_{1}*X_{2}–\;0.1*X_{1}{}^{2}$$

In Figure 2(b), it can be observed that as X_1_ increased, the DL gradually increased to its maximum point and then decreased just like an umbrella asymptotic curve. This result can be explained as follows, increasing Pt amount within a certain range would lead to subsequent higher drug incorporation. However, once the amount of feeding Pt exceeded the capability of the polymeric micelles, DL would decrease. As for X_2_, we observed that the influence of hydration volume on DL was similar to that on EE (Figure 2(a,b)). The elevation of PBS volume resulted in higher drug incorporation as well as higher DL.

The data in Table 1 showed that the mean HD of prepared polymeric micelles ranged from 74.5 to 169.2 nm. When HD was considered as the dependent response, good correlation was obtained between predicted and observed value as revealed by an adjusted R^2^ value of 0.91. The ANOVA (Table 2) results showed that in this case, independent X_1_, X_2_ and polynomial term X_1_X_2_, X_1_^2^ were significant model terms with p-values of <0.05. The following regression equation of the fitted quadratic model could explain the effect of X_1_ and X_2_ on HD:

(9) $$\text{HD}\;\left(\text{nm}\right)\;=\;63.8\;+\;10.0*X_{1}–\;2.6*X_{2}+\;0.5*X_{1}*X_{2}–\;0.5*X_{1}{}^{2}-\;0.1*X_{2}{}^{2}$$

As shown in Figure 2(c), at a high level of X_2_, the HD increased with the increase of X_1_. Meanwhile, the HD increased to its maximum point and then decreased with increasing X_1_ at a low level of X_2_. This phenomenon was expected because incorporation of adequate Pt molecules into the hydrophobic cores would increase the volume of the polymeric micelles (Kheiri Manjili et al., 2017b). And as mentioned above, increasing the value of X_2_ might enhance the entrapment ability of the drug leading to larger HD.

**Figure 3.**
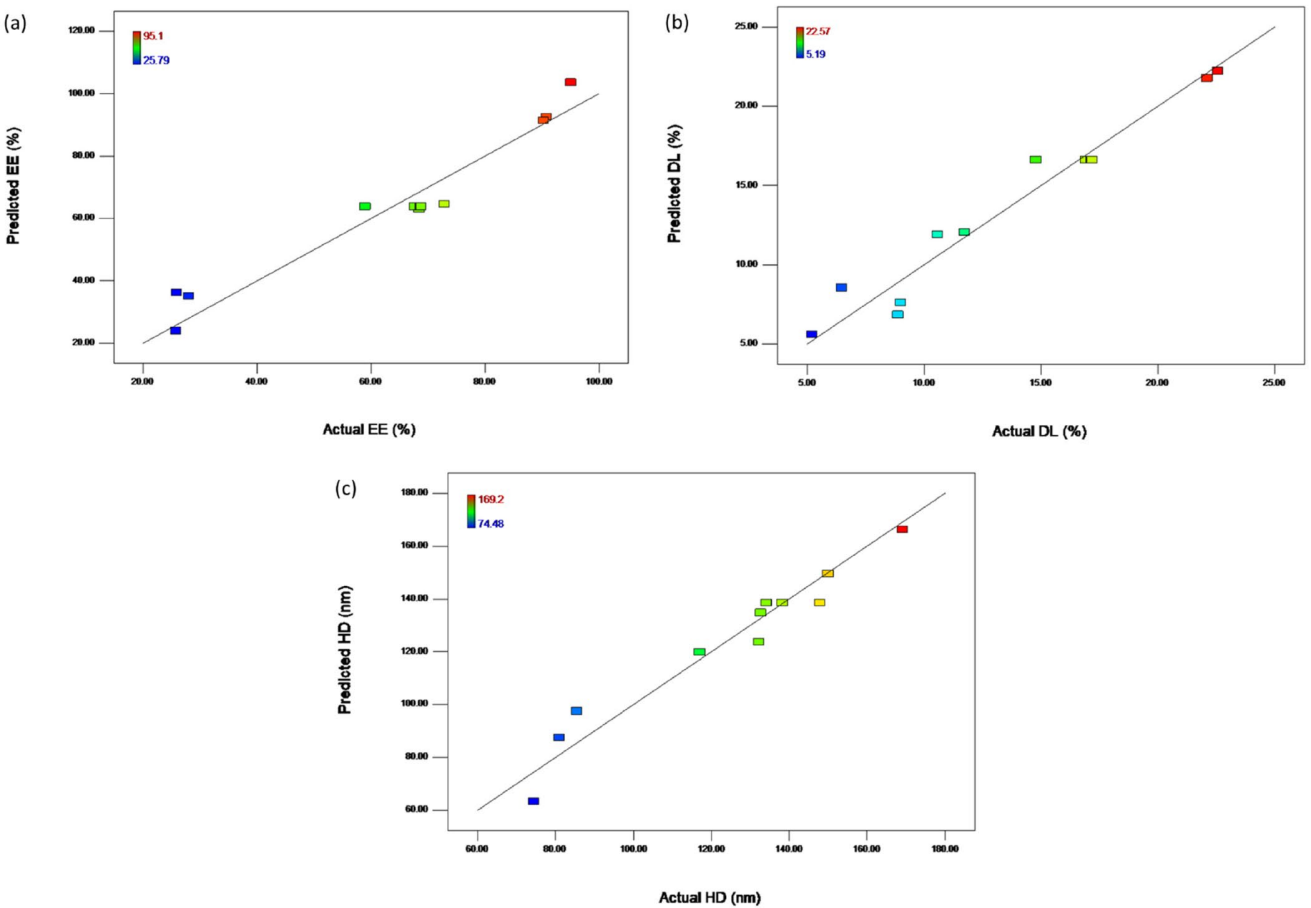
Correlation of actual (a) EE, (b) DL, (c) HD and values predicted by the response surface model. EE, entrapment efficiency; DL, drug loading and HD, hydrodynamic diameter.

The fitness and accuracy of the above three predicted models were considered based on the data of ANOVA (Table 2).The p value of each model was less than 0.001, indicating that the model terms were statistically significant. Coefficient of variation (CV) was calculated as a measure of how precise and reliable the model was (<10% is considered highly favorable) and the values were found to be 10.4%, 11.2% and 7.0%, respectively. Adequate precision (AP) gives the signal to noise ratio (value >4 is desirable). The responses showed AP > 4, demonstrating that the developed models were suitable. The lack of fit value of each response model was greater than 0.05, indicating that this value was not significant relative to the pure error. The model reliability was also estimated based on the data of fitted line plots of predicted versus actual (see Figure 3). A linear distribution implied the intimate closeness between the experimental and predicted values for the dependent responses, showing that the models were well-fitted.

### 3.2. Optimization of the preparation

In this study, F-Pt/M was successfully prepared by using thin-film hydration method and optimized based on the criteria for obtaining the maximum EE and DL, while minimizing HD. After setting the acceptable minimum values (lower limits) of EE, DL and HD as 90%, 10% and 20 nm, respectively and the maximum values (upper limits) as 100%, 40% and 200 nm, respectively, the optimization procedure was performed automatically using the Design Expert 8.0.6. The optimum F-Pt/M formula was determined as 8.3 mg of Pt (X_1_), 15.0 mL of hydration PBS solution (X_2_), 30.0 mg of PEG_2000_-PCL_2000_ and 3.0 mg of Folate-PEG_3400_-PCL_2000_. In terms of model validation, the recommended optimal independent X_1_, X_2_ variables and predicated responses were verified by performing the experiment in triplicate under the calculated optimal conditions. Table 3 exhibits the predicted and observed values of the responses at the optimal independent variables level according to the fitted polynomial models for the response variables. The results demonstrated that the experimental values were in good agreement with the predicted ones, indicating the practicability of the models in optimizing Pt loaded polymeric micelles formulation. It’s worth noting that zeta potential of nanoparticles, as an important parameter, should be included in the optimization data (Rana et al., 2020; Gul et al., 2022). However, the zeta potential values didn’t change much within our experimental limits. Hence, it wasn’t taken as a prescriptive criterion in the present study.

**Table 3.** Experimental and predicted values of each response based on the optimal preparation formulation for folate modified pterostilbene loaded micelles.

Responses	Values	
	Experimental	Predicted
EE (%)	98.6 ± 0.8	100.00
DL (%)	18.3 ± 0.6	19.0
HD (nm)	133.2 ± 1.2	126.6

### 3.3. Characterizations of folate modified pterostilbene loaded PEG_2000_/PEG_3400_-PCL_2000_ micelles

#### 3.3.1. Hydrodynamic diameter and zeta potential

**Figure 4.**
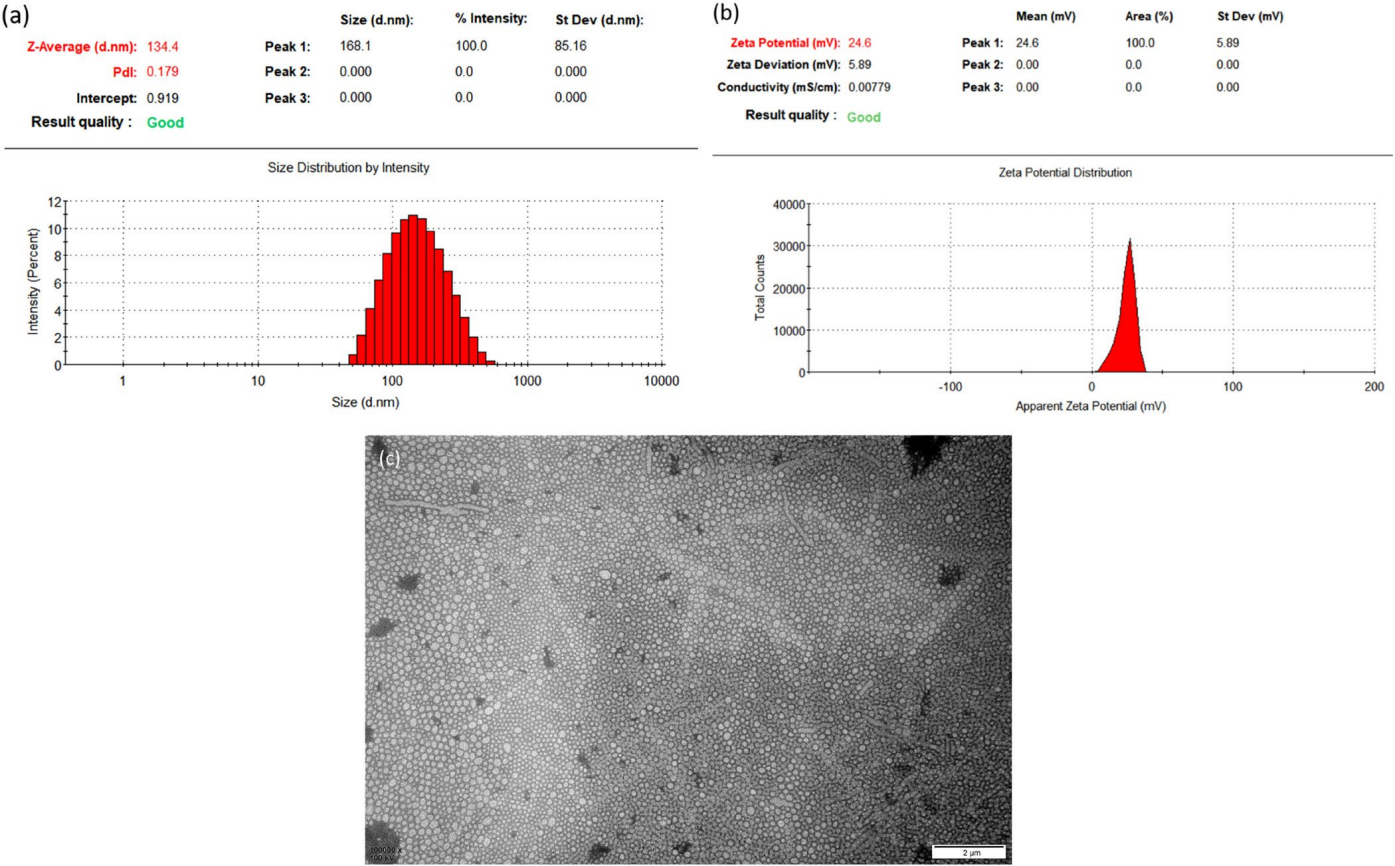
Representative hydrodynamic diameter and polydispersity index (a), zeta potential (b) analysis and TEM photomicrograph (c) of the optimized folate modified pterostilbene loaded polymeric micelles.

The mean hydrodynamic diameter of optimal F-Pt/M was 133.2 ± 1.2 nm with a low PDI of 0.2 ± 0.02 and a unimodal particle size distribution curve (see Figure 4(a)). Hydrodynamic diameter/Particle size, one of the most important physicochemical features, contributed to the passive targeting ability and anticipant tissue distribution of nano-carries. Micelles with sizes between 20 and 200 nm usually possess better biological characteristics, such as the ability to overcome the BBB and greater internalization into tumor cells (Betzer et al., 2017; Luiz et al., 2021). Therefore, this developed F-Pt/M formulation with hydrodynamic diameter of 133.2 nm was expected to enhance the penetration of Pt through the BBB in facilitating the treatment of brain diseases.

The average zeta potential of F-Pt/M was 24.6 ± 1.0 mV (see Figure 4(b)). To obtain a physically stable nano-system, the absolute value of zeta potential should be at least 20 mV for sterically stabilized or 30 mV for electrostatic systems (Juyang & Wolf, 2021). The hydrophilic interactions between hydrophilic PEG chains shell of constructed F-Pt/M, steric hindrance and electrostatic repulsion could prevent the aggregation of the micelles and keep the colloid nano-system stable (Zhao et al., 2012a).

#### 3.3.2. TEM observation

TEM photomicrograph of the optimal F-Pt/M formulation was illustrated in Figure 4(c). It was clear that the formed micelles were fairly dispersed in aqueous medium and developed homogeneous small-sized spherical micellar structures with a narrow size distribution. The mean particle size observed by TEM looked smaller than that determined by LLS technique (Figure 4(a)). The particle size measured by LLS reflected the hydrodynamic diameter of micellar nanoparticles. The hydration of the PEG segment of polymeric PEG-PCL material could result in an increased particle size. Oppositely, the nanoparticle size observed by TEM reflected the shrinkage of such micellar nanoparticles during drying process in the TEM preparation (Liu et al., 2014).

#### 3.3.3. DSC analysis

**Figure 5.**
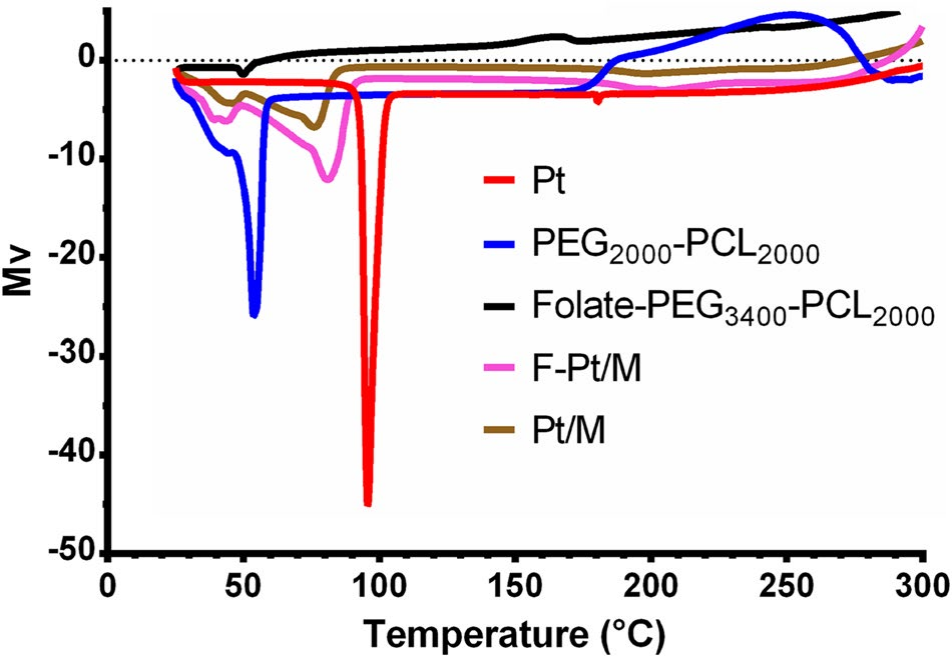
DSC thermograms of Pt, PEG_2000_-PCL_2000_, Folate-PEG_3400_-PCL_2000_, Pt/M, and the optimized polymeric micelles F-Pt/M. Pt, pterostilbene; Pt/M, lyophilized pterostilbene loaded micelles; F-Pt/M, lyophilized folate modified pterostilbene loaded micelles.

DSC analysis was employed to make available information on the drug-copolymer relationship and the physical changes happened on the drug or polymer can be considered by means of the thermal analysis. The DSC study was done for Pt, excipients (PEG_2000_-PCL_2000_ and Folate-PEG_3400_-PCL_2000_), Pt/M and for F-Pt/M candidate formulation. Figure 5 showed the DSC thermogram of Pt with a sharp characteristic endothermic peak at 95.9 °C, indicating its crystalline state, which was not observed in micellar particulate Pt. it could be concluded that the Pt in the micelles was in an amorphous or solid solution state or in a disordered crystalline phase (Xie et al., 2007). Concerning thermograms of PEG_2000_-PCL_2000_ and Folate-PEG_3400_-PCL_2000_, endothermic peaks were detected at 53.1 °C and 50.0 °C, respectively, indicating the melting of the crystalline PCL segment of copolymer (Kheiri Manjili et al., 2017a). Regarding the DSC thermogram of lyophilized Pt/M, two peaks were observed at 43.4 °C and 76.1 °C. The thermogram of lyophilized F-Pt/M displayed a pattern similar to that of Pt/M, with two peaks at 39.0 °C and 80.8 °C. The disappearance of the characteristic endothermic peak of Pt (95.9 °C) indicated the successful entrapment of the drug in the developed PM or F-Pt/M.

#### 3.3.4. Entrapment efficiency (EE) and drug loading (DL)

The EE and DL values are important indexes for drug delivery systems. The average EE and DL of the optimized F-Pt/M formulation were 98.6 ± 0.8% and 18.3 ± 0.6%, respectively. The solubility of Pt in water was increased to be about 545.6 µg/mL, which was about 26.0 times that of crude Pt in water (about 21 µg/mL) (Liu et al., 2020). The results indicated that hydrophobic Pt molecules could be readily incorporated into the hydrophobic core of the developed F-Pt/M through hydrophobic interactions and significantly enhance the solubility of Pt in aqueous solution.

### 3.4. In vitro drug release

**Figure 6.**
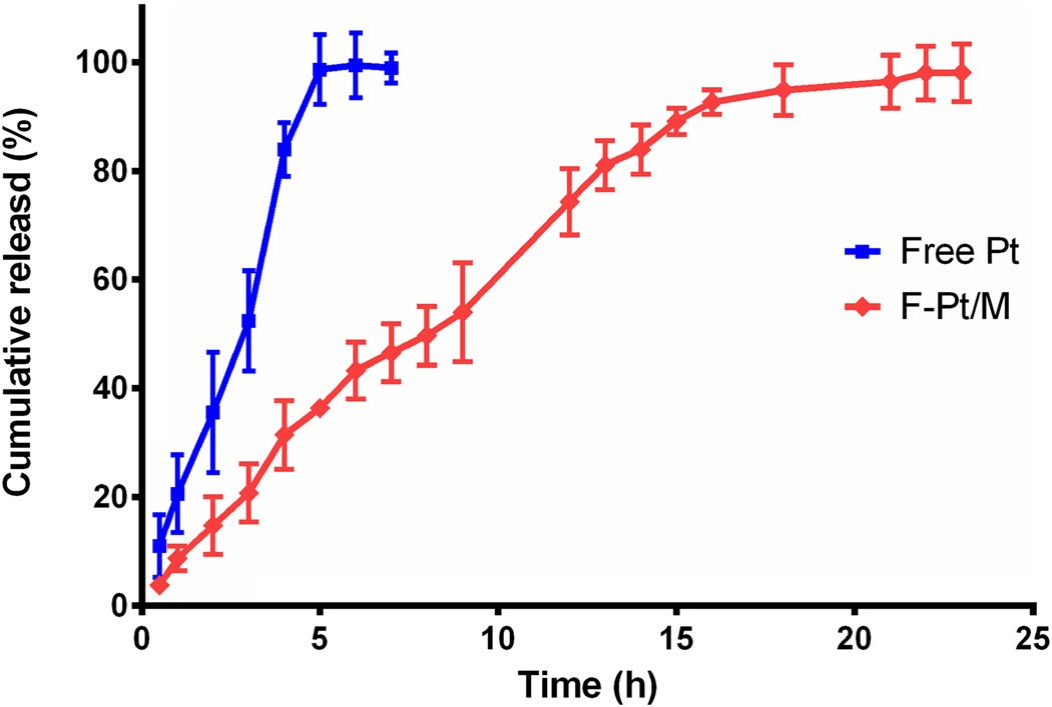
In vitro release profiles of pterostilbene from free Pt and F-Pt/M in PBS (pH 7.4) containing 0.3% Tween 80. Free Pt, free pterostilbene acetone solution; F-Pt/M, folate modified pterostilbene loaded micelles.

The in vitro release curves of free Pt and Pt loaded F-Pt/M were listed in Figure 6. An obvious initial burst release was shown in the release profile of free Pt acetone solution, and almost all the drug was released within the first 5 h. However, the F-Pt/M formulation could release Pt over 20 h without dramatic initial burst, and about 98% of Pt was released ultimately. These results indicated that Pt loaded micelles could slow down the release rate of Pt with a sustained release behavior.

Drug release from polymeric micelles is a complex process involving drug diffusion, relaxation of the polymer chain, or erosion. In order to study release behavior of Pt from F-Pt/M, the release curve was fitted with zero-order, first-order and Higuchi release models using Origin 2019b Software. The results showed that adjusted R^2^ value for First-order model was 0.987, being the highest among the three models (adjusted R^2^ values for zero-order and Higuchi models were 0.933 and 0.977, respectively). This meant the First-order model was suitable for describing the drug release behavior from F-Pt/M, demonstrating that the release rate of Pt from F-Pt/M was only related to the concentration of residual drug in the polymeric micelles.

To illustrate the release mechanism, Pt release data was fitted with Korsmeyer-Peppas model. In this model, the n value is an important release index representing various release mechanisms. For spherical polymeric controlled delivery systems, such as micelles, when n value is ≤0.43, a classical Fickian diffusion process can explain the drug release mechanism, which could be observed in non-swelling systems. If *n* ≥ 0.85, the drug release mechanism is Case II transport, which refers to the erosion/relaxation of the polymer chains. Values of n between 0.43 and 0.85 imply an Anomalous transport (non-Fickian diffusion) process, which is intermediate to Fickian diffusion and Case II transport (Zuo et al., 2014; Wei et al., 2018). In our study, the n value was 0.749 which was between 0.43 and 0.85, indicating that the drug release process was an Anomalous transport-controlled manner.

### 3.5. In vitro cytotoxicity and cellular uptake

#### 3.5.1. Cytotoxicity assay

**Figure 7.**
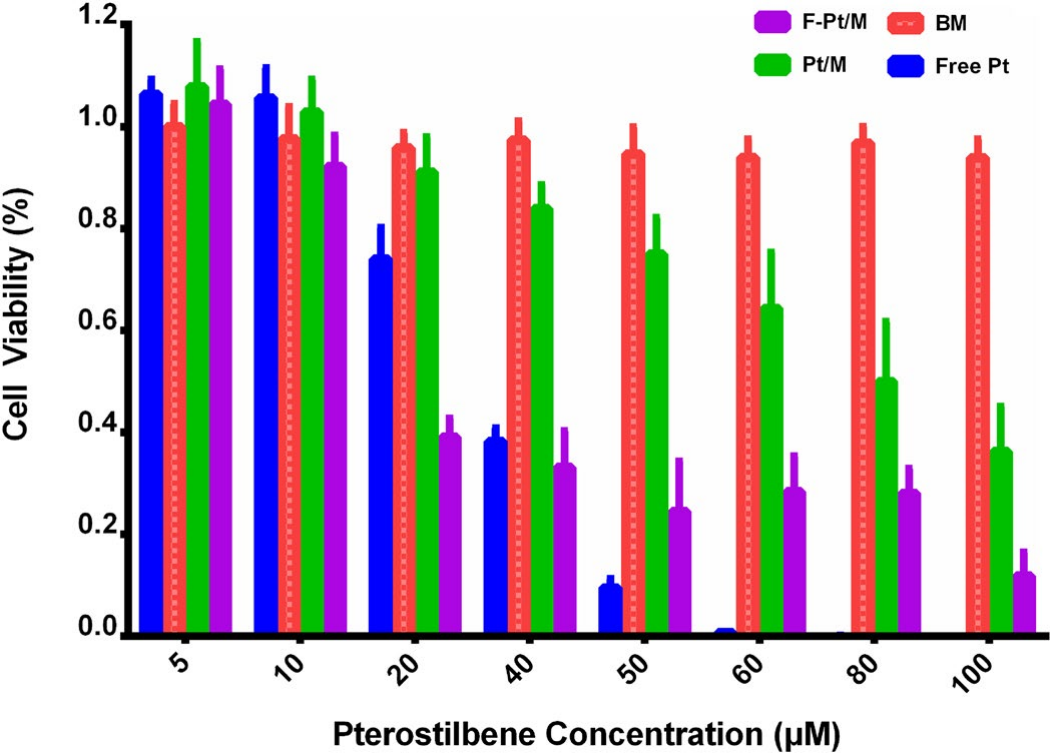
In vitro cytotoxicity of free pterostilbene solution (free Pt), pterostilbene loaded micelles (Pt/M), folate modified pterostilbene loaded micelles (F-Pt/M) and blank micelles (BM) against A172 cells after treatment for 48 h.

**Figure 8.**
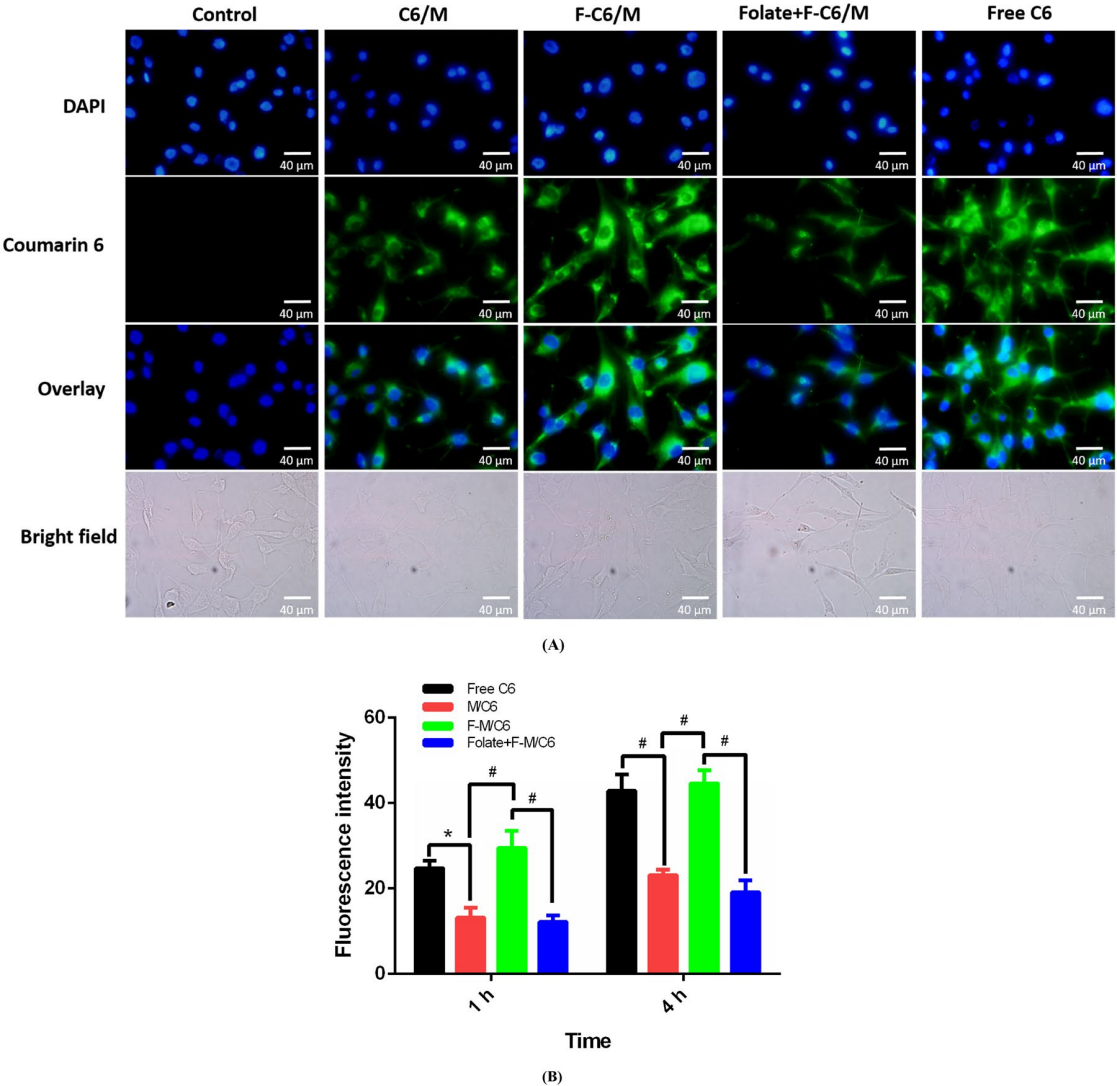
Cellular uptake study of various C6 formulations in A172 cells. (A) Fluorescence microscope images of cells incubated with free C6, C6/M and F- C6/M in media with or without free folate at 37 °C for 4 h. For folate + F- C6/M group, A172 cells were pretreated with excess of free folate at 10 μg/mL for 30 min, followed by incubation with F- C6/M. The concentration of C6 was 100 ng/mL for all formulations. (B) Intensity profile of C6 fluorescence based on spectrophotometer measurement (*n* = 3, *p < 0.01, #*p* < 0.001). Green and blue represent fluorescence of C6 and DAPI, respectively. Enhanced cellular uptake by folate modification was significantly blocked in the presence of free folate. C6, coumarin-6; C6/M, coumarin-6 loaded micelles; F-C6/M, folate modified coumarin-6 loaded micelles.

The in vitro cytotoxicity of free Pt, Pt/M, F-Pt/M and corresponding BM against A172 cells was investigated by CCK-8 method (Figure 7). For BM, cell viabilities were around 100%, indicating that it had no obvious cytotoxicity to A172 cells during the test period, confirming any cytotoxicity of Pt loaded micelles observed was thus mainly due to the effects of the released drug alone. The cytotoxicity of free Pt, Pt/M and F-Pt/M was in a dose-dependent manner. With the increase of drug concentration, cell viability decreased, showing that higher drug concentration is essential for the drug to effectively inhibit tumor cells. The IC_50_ values for free Pt, Pt/M and F-Pt/M were 24.43 μM, 82.02 μM and 22.33 μM, respectively. When Pt was encapsulated into folate modified F-Pt/M, its cytotoxicity to A172 cells was increased by 2.67 times compared to Pt/M after 48 h of incubation. This could be ascribed to the higher ligand-receptor mediated cellular uptake of the F-Pt/M. The in vitro cytotoxicity results of Pt/M and F-Pt/M were in accordance with the data of the cellular uptake assay (Figure 8). The IC_50_ value of F-Pt/M was similar to that of free Pt, implying that, unlike Pt/M, the encapsulation of Pt into F-Pt/M would not affect the therapeutic efficacy of the drug. However, at high drug concentrations (>50 μM), the cell cytotoxicity of free Pt was significantly greater than both of the Pt/M and F-Pt/M (*p* < 0.05). Hailu Yao et al. (Yao et al., 2020) prepared cantharidin loaded mPEG-PLGA micelles (mPEG-PLGA-CTD). In their study, the free cantharidin also had greater cytotoxicity to L-02 cells than mPEG-PLGA-CTD at higher drug concentrations. The reason could be mostly attributed to different cell uptake pathways of free drug molecules and micelles, and the release manner of drug-loaded micelles. Therefore, it is clear that the F-Pt/M formulation exhibited higher cytotoxicity than the Pt/M formulation, which showed that folate receptor mediated endocytosis could facilitate the cellular uptake and hence strengthen the cytotoxic effect of the drug-loaded polymeric micelles.

#### 3.5.2. In vitro cellular uptake

In order to study the penetration of the micelles into the cells and the targeting effect of the micelles modified with folate, cellular uptake of the F-C6/M was performed using human glioblastoma A172 cells. Folate, as an attractive target ligand, can be easily recognized by folate receptor that is overexpressed in a large number of cancer cells (Wang et al., 2012). Thus, we introduced Folate-PEG-PCL into the preparation of Pt loaded micelles to promote the cellular internalization by receptor-mediated endocytosis and increase their therapeutic efficiency. Figure 8(a) showed the uptake of various C6 formulations into A172 cells monitored by fluorescence microscope. The results demonstrated that free C6 could easily enter into cells and led to intense intracellular fluorescence possibly due to its highly hydrophobic nature. F-C6/M group showed brighter intracellular green C6 fluorescence compared with C6/M group, suggesting that the folate could markedly facilitate and promote the entry of micelles into cells. Pretreatment of cells with free folate obviously inhibited the uptake of F-C6/M in A172 cells. F-C6/M had a 2.43- and 2.34-fold uptake relative to that of free folate pretreated C6/M (Figure 8(b)) after 1 h and 4 h incubation at 37 °C, indicating that enhanced cellular uptake by folate modification could be blocked in the presence of free folate. These results directly illustrated that the cellular uptake of the F-C6/M can be boosted by folate receptor mediated endocytosis process, which were consistent with previous studies regarding folate receptor dependent cellular uptake of folate-conjugated nanoparticles for anticancer therapy (Liu et al., 2010; Zhao et al., 2012b; Kucheryavykh et al., 2019).

### 3.6. Hemolytic toxicity measurement

**Figure 9.**
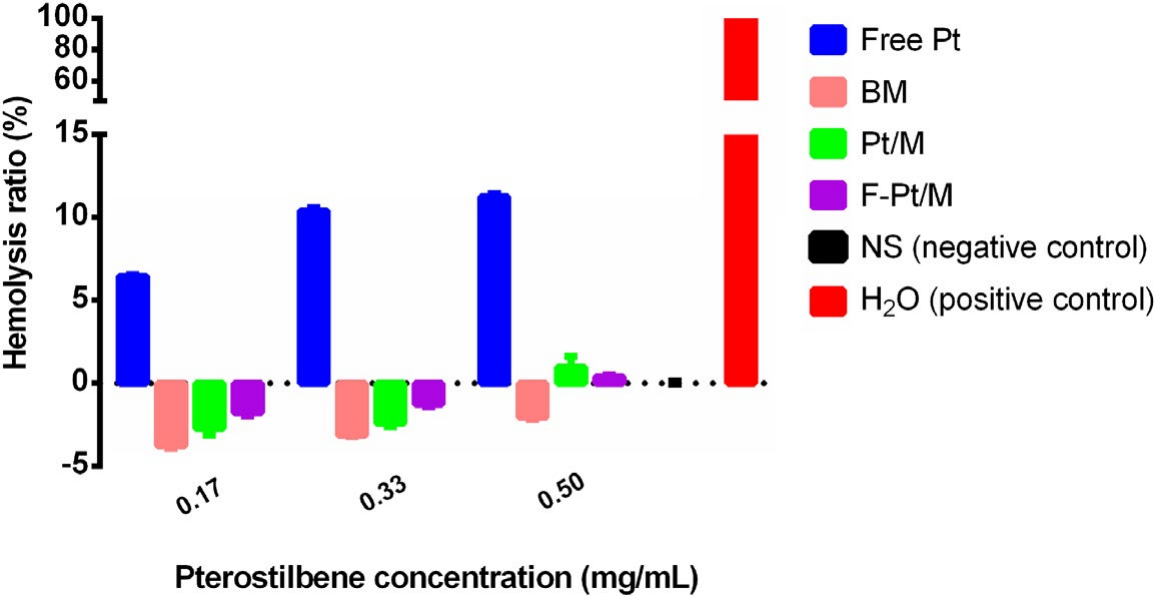
Hemolytic test on free Pt and different micelle formulations. The data represent the mean ± SD (*n* = 3). Free Pt, free pterostilbene solution; BM, blank micelles; Pt/M, pterostilbene loaded micelles; F-Pt/M, folate modified pterostilbene loaded micelles; NS, normal saline.

To validate the safety of F-Pt/M in the case of in vivo application, hemolysis assay was performed. Blood compatibility of polymeric micelles is an important characteristic for intravenous administration (Valenzuela-Oses et al., 2017). If the HR was less than 5%, the formulation could be regarded as non-hemolytic and further consideration could be given to explore its use through intravenous route (Richter et al., 2010). It was found that the values of HR increased accordingly with the concentration of free Pt (Figure 9). The HR was 6.46%, 10.39% and 11.23% for low concentration of 0.17 mg/mL, medium concentration of 0.33 mg/mL and high concentration of 0.5 mg/mL, respectively. However, the HRs were found to be less than 5% for all micellar preparations, including blank micelles, drug-loaded Pt/M and folate modified F-Pt/M, suggesting that blank micelles as well as F-Pt/M had negligible hemolytic toxicity within the studied concentration range.

### 3.7. In vivo brain distribution study in rats

**Figure 10.**
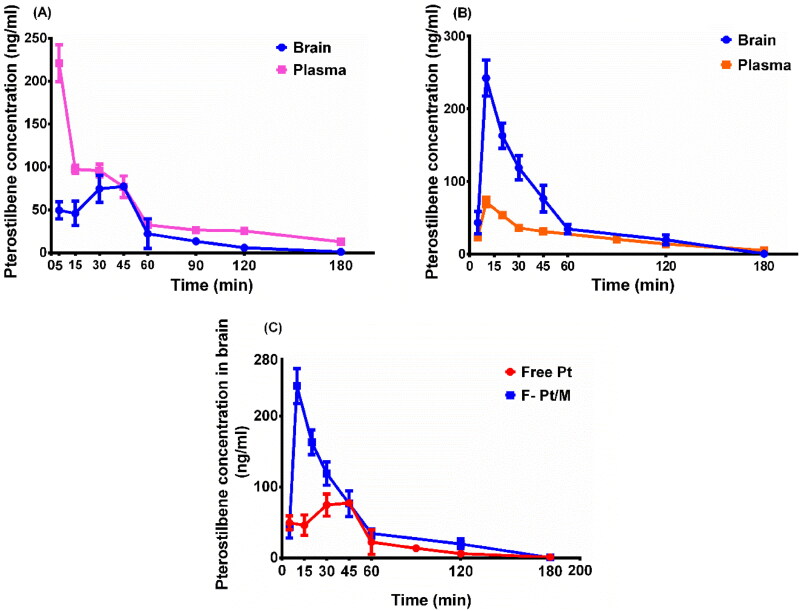
In vivo pterostilbene concentration vs. time profiles of the different samples (brain and plasma) after a single intravenous administration of (A) free pterostilbene solution (free Pt), (B) folate modified pterostilbene loaded micelles (F-Pt/M) at a dose of 3 mg/kg in rats. And (C) pterostilbene concentration in brain vs. time profiles of free Pt and F-Pt/M. Serial brain/blood samples were collected at 5, 15, 30, 45,60, 90,120 and 180 min. The data represent the mean ± SD (*n* = 3).

The brain distribution of Pt formulated in the F-Pt/M formulation was investigated in comparison with free Pt solution as control after i.v. administration (Figure 10). The key brain distribution pharmacokinetic parameters including AUC_0–t_, the area under the concentration-time curve, t_1/2_, elimination half-life, C_max_, the maximum concentration of Pt and T_max_, the time to C_max_ were analyzed and listed in Table 4. Results showed that Pt had a statistically significant increase in brain C_max_ and AUC_0–t_ when it was encapsulated in F-Pt/M. The C_max_ in brain for F-Pt/M was 242.33 ± 24.58 ug/L which was about 2.1 times higher than that of free Pt. The AUC_0-t_s of Pt in the brain were found to be 4369.66 ± 289.88 μg/L min and 8844.05 ± 894.12 μg/L min for free Pt and F-Pt/M, respectively (Table 4). Meanwhile, F-Pt/M showed a shorter T_max_ in the brain compared to that of free Pt (*p* < 0.05), demonstrating that F-Pt/M not only increased drug distribution in the brain, but also improved the transport rate of Pt through BBB. The folate conjugation was beneficial to increase the uptake of Pt into the brain through receptor-mediated endocytosis. Furthermore, highly lipophilic nature of F-Pt/M could contribute to greater brain uptake compared to free Pt. To examine the blood-brain barrier penetration of Pt, BBB penetration (P) values were calculated. F-Pt/M resulted in an almost 4-fold increase in Pt permeation into the brain in comparison with free Pt. Further, tissue targeting index was calculated to determine targeting of F-Pt/M to the brain. The tissue targeting index was found to be 4.89, which indicated improved brain targeting was achieved using the folate modified F-Pt/M formulation. We didn’t investigate the effect of FA density on the targeting ability of our polymeric micelles. Huile Gao group reported that the strong binding affinity of ligand with receptors could inhibit the transcytosis through BBB and the cleavable ligand modification could improve the targeting delivery efficiency (Ruan et al., 2018; Lei et al., 2021; He et al., 2022). Thus, further research still needs to be done in the following work.

**Table 4.** Pharmacokinetic parameters of pterostilbene following single intravenous administration of free pterostilbene aqueous solution (free Pt) and pterostilbene loaded micelles (F-Pt/M) at a dose of 3 mg/kg, in rats. Values listed as mean ± SD (*n* = 3).

Pharmacokinetic parameters	free Pt		F-Pt/M	
	Plasma	Brain	Plasma	Brain
AUC_0-t_ (μg/L min)	10107.80 ± 976.65	4369.66 ± 289.88	4181.75 ± 142.20**	8844.05 ± 894.12**
t_1/2_ (min)	114.25 ± 29.23	22.38 ± 2.84	54.27 ± 10.10	35.96 ± 17.61
T_max_ (min)	5	35 ± 8.66	10	10*
C_max_ (ug/L)	220.98 ± 21.55	78.13 ± 13.95	71.57 ± 6.43**	242.33 ± 24.58**

AUC_0–t_, the area under the concentration-time curve; t_1/2_, elimination half-life; C_max_, the maximum concentration of Pt; T_max_, the time to C_max_.

*p < 0.05, compared to the free Pt.

**p < 0.01, compared to the free Pt.

## 4. Conclusion

In conclusion, a novel nano polymeric micellar drug delivery system named F-Pt/M was developed, which was composed of Pt (as model drug), folate (as targeting ligand) and PEG_2000_/PEG_3400_-PCL_2000_ (as self-assembled material) to enhance the penetration of Pt across the BBB. The F-Pt/M with high drug loading capacity (18.3%) and entrapment efficiency (98.6%) were prepared using thin-film hydration method. Pt was released in a sustained manner with no dramatic initial drug burst, offering the possibility to continually fight against target cells. In vitro cytotoxicity assays confirmed that folate conjugated F-Pt/M had a significantly higher cytotoxicity against FR-overexpressing A172 cells than that of Pt/M without folate modification. Additionally, coumarin-6 loaded F-C6/M further showed higher efficient endocytosis abilities when compared to non-targeted C6/M, implying the effective cellular accumulation of folate targeted micelles via receptor-mediated endocytosis. Brain targeting and brain distribution study in rats illustrated that F-Pt/M had higher drug targeting index (DTI) and BBB penetration (P) values in comparison with free Pt, suggesting that F-Pt/M could significantly improve the efficiency in delivering Pt to the brain. Based on these findings, it can be concluded that F-Pt/M might have the potential to be developed as an attractive micellar system for effective chemotherapy against some brain diseases.

## Abbreviations

ANOVA: one-way analysis of variance
AUC_0-t_: area under plasma concentration-time curve
BBB: blood-brain barrier
BM: blank micelles
C6: coumarin-6
C6/M: coumarin-6 loaded polymeric micelles (without folate modification)
C_max_: maximum drug concentration
CNS: central nervous system
DSC: differential scanning calorimetry
DTI: drug targeting index
EE: entrapment efficiency, DL, drug loading
FA: folic acid
F-C6/M: folate modified coumarin-6 loaded polymeric micelles
F-Pt/M: folate modified pterostilbene loaded polymeric micelles
FR: folate receptor
HD: hydrodynamic diameter
HR: hemolysis ratio
MPEG-PCL: methoxy poly(ethylene glycol) block PCL
MTBE: methyl tertiary butyl ether
PDI: polydispersity index
Pt: pterostilbene
Pt/M: pterostilbene loaded polymeric micelles (without folate modification)
RBC: red blood cells
RSM: response surface methodology
t_1/2_: elimination half-life
TEM: transmission electron microscopy
T_max_: the time to C_max_
